# Preparation and Evaluation of a Self-Emulsifying Drug Delivery System for Improving the Solubility and Permeability of Ticagrelor

**DOI:** 10.1021/acsomega.3c08700

**Published:** 2024-02-21

**Authors:** Anam Aziz, Muhammad Zaman, Mahtab Ahmad Khan, Talha Jamshaid, Muhammad Hammad Butt, Huma Hameed, Muhammad Shafeeq Ur Rahman, Qurat-ul-Ain Shoaib

**Affiliations:** † Faculty of Pharmaceutical Sciences, University of Central Punjab, Lahore 54000, Pakistan; ‡ Faculty of Pharmacy and Alternative Medicine, The Islamia University Bahawalpur, Bahawalpur 63100, Pakistan; § Department of Medicinal Chemistry, Faculty of Pharmacy, Uppsala University, 75123 Uppsala, Sweden; ∥ Akhtar Saeed College of Pharmaceutical Sciences, Lahore 54000, Pakistan

## Abstract

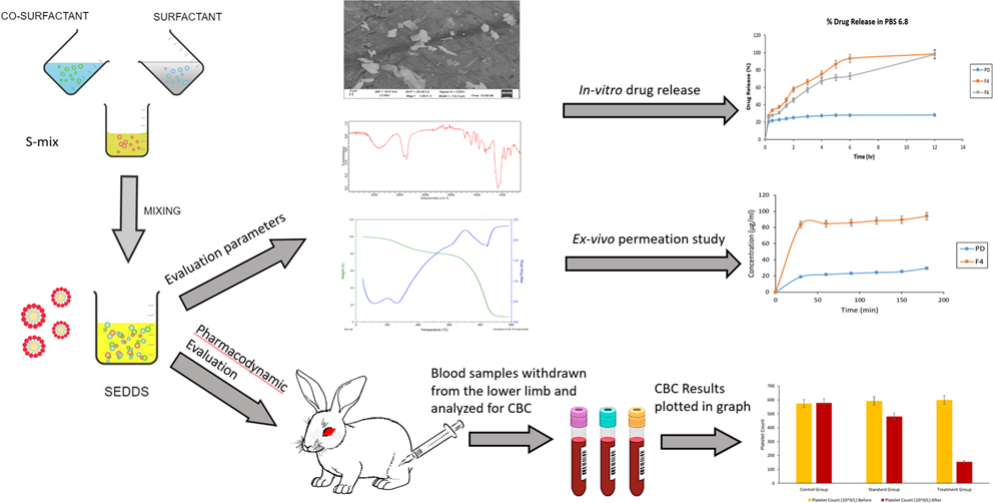

Ticagrelor (TCG) is a BCS class IV antiplatelet drug used to prevent platelet aggregation in patients with acute coronary syndrome, having poor solubility and permeability. The goal of this study was to develop a self-nanoemulsifying drug delivery system (SNEDDS) of TCG to improve its solubility and permeability. The excipients were selected based on the maximum solubility of TCG and observed by UV spectrophotometer. Different combinations of oil, surfactant, and co-surfactant (1:1, 2:1, and 3:1) were used to prepare TCG-SNEDDS formulations, and pseudo-ternary phase diagrams were plotted. The nanoemulsion region was observed. Clove oil (10–20%), Tween-80 (45–70%), and PEG-400 (20–45%) were used as an oil, surfactant, and co-surfactant, respectively. The selected formulations (F1, F2, F3, F4, F5, and F6) were analyzed for ζ potential, polydispersity index (PDI), ζ size, self-emulsification test, cloud point determination, thermodynamic studies, entrapment efficiency, Fourier transform infrared (FTIR) spectroscopy, X-ray diffraction (XRD), differential scanning calorimetry (DSC), thermogravimetric analysis (TGA), scanning electron microscopy (SEM), *in vitro* dissolution, *ex vivo* permeation, and pharmacodynamic study. The TCG-SNEDDS formulations exhibited ζ potential from −9.92 to −6.23 mV, a ζ average of 11.85–260.4 nm, and good PDI. The *in vitro* drug release in phosphate buffer pH 6.8 from selected TCG-SNEDDS F4 was about 98.45%, and F6 was about 97.86%, displaying improved dissolution of TCG in 0.1 N HCl and phosphate buffer pH 6.8, in comparison to 28.05% of pure TCG suspension after 12 h. While the *in vitro* drug release in 0.1 N HCl from F4 was about 62.03%, F6 was about 73.57%, which is higher than 10.35% of the pure TCG suspension. In *ex vivo* permeability studies, F4 also exhibited an improved apparent permeability of 2.7 × 10^–6^*versus* 0.6708 × 10^–6^ cm^2^/s of pure drug suspension. The pharmacodynamic study in rabbits demonstrated enhanced antiplatelet activity from TCG-SNEDDS F4 compared to that from pure TCG suspension. These outcomes imply that the TCG-SNEDDS may serve as an effective means of enhancing TCG’s antiplatelet activity by improving the solubility and permeability of TCG.

## 1 Introduction

There has been a growing prevalence of pharmacological compounds that have been newly identified, exhibiting a reduced ability to dissolve in water and subsequently leading to decreased absorption rates when administered orally. Poor water solubility affects around 35–40% of all novel chemical entities identified, and the administration of these drugs is typically linked with inadequate bioavailability, considerable inter- and intrasubjective variability, and the lack of dosage proportionality.^1^ New chemical entity (NCE) characteristics changed toward larger molecular weight and increased lipophilicity, resulting in lower water solubility. Many drug candidates fail to reach the market due to poor aqueous solubility, although this indicates potential pharmacodynamic action. In addition, drugs with low water solubility are sometimes supplied at far higher individual doses than necessary to achieve desired plasma concentrations.^2,3^ Micronization, lipid-based systems, salt formation, use of metastable polymorphs, pH alteration of the microenvironment, formation of solute–solvent complexes, solid dispersion, and molecular encapsulation with cyclodextrins and solvent deposition, among other pharmaceutical methodologies, aim to address poor solubility, permeability, and bioavailability of insoluble drug.^4−7^ Lipid-based systems, including nanoemulsion, microemulsion, self-emulsifying drug delivery system, and other related methods, have significant potential as a promising technology.^8^

A self-emulsifying drug delivery system (SEDDS) is defined as “an isotropic and thermodynamically stable system comprising of drug, oils, surfactants, and co-surfactant or co-solvents”. SEDDS are given in the oil-in-water form, and when it comes in contact with stomach content, they form coarse, micro-, or nano-size emulsion based on the contents and formulation process. The primary mechanism that supports SEDDS in increasing the dissolution rate is the natural production of an emulsion within the GI system due to gentle agitation caused by stomach motility. The decline in the size of the droplets results in an expansion in the interfacial area, hence promoting drug absorption. Consequently, SEDDS enhances the solubility of hydrophobic drugs in aqueous environments. The use of SEDDS has been shown to enhance the absorption of drugs *via* improvements in drug solubility, permeability, and lymphatic uptake.^9,10^

Ticagrelor (TCG), C23H28F2N6O4S, is a solid, white crystalline powder exhibiting 10 μg/mL solubility in water. It has a molecular weight of about 522.6 g/mol.^11^ It belongs to an antiplatelet agent class known as cyclopentyltriazolopyrimidines, which inhibits platelet aggregation by acting directly onto the P2Y12 receptor without the need for metabolic activation. In patients suffering from acute coronary syndrome (ACS) or a prior myocardial infarction (MI), TCG prevents the production of occlusive thromboses, intended to lower the risk of cardiovascular mortality, myocardial infarction, and ischemic stroke. The oral bioavailability of TCG is 36% and has a plasma half-life of about 8 h as compared to the active metabolite of TCG, *i.e.*, AR-C124910XX, which has a plasma half-life of about 12 h.^12^ It can be given either once or twice a day, having a moderate duration of action and a broad therapeutic index because the large single doses can be very well tolerated.

According to comparative trials, TCG has been shown to provide higher and more steady degrees of platelet aggregation inhibition and a favorable trend in lowering the risk of myocardial infarction compared to clopidogrel without elevating the risk of severe bleeding. Ticagrelor is not a prodrug, unlike clopidogrel,^12,13^ and shows extremely little solubility at all pH levels. However, it falls under the biopharmaceutical classification system (BCS) of class IV because of its limited intestinal membrane permeability and poor solubility. Few studies have been conducted, despite the recent reporting of the formulations to increase the bioavailability and antiplatelet effect of ticagrelor, such as solid dispersion^14^ and co-crystallization.^15^

The primary aim of this research is to formulate self-emulsifying drug delivery systems (SEDDS) for ticagrelor, a drug falling under the Biopharmaceutics Classification System (BCS) class IV category. The purpose is to enhance the solubility and permeability attributes of the drug.

## 2 Materials and Methods

### 2.1 Materials

Ticagrelor (TCG) was a gift from CCL Pharmaceuticals (Lahore, Pakistan, Punjab). Clove oil, silicon oil, lavender oil, olive oil, oleic acid, Tween-20 (polyoxyethylenesorbitan monolaurate), triethanolamine (Sterolamide), Tween-80 (polyoxyethylenesorbitan monooleate), Transcutol-P (diethylene glycol monoethyl), PEG-400 (polyethylene glycol-400), and methanol were kindly provided by Sigma-Aldrich. Distilled water was utilized throughout the course of the experiment.

### 2.2 Methods

#### 2.2.1 Linearity Curve Determination of Ticagrelor

The standard stock solution was prepared by taking 10 mg of the ticagrelor drug and placing it in a 100 mL volumetric flask. To achieve a concentration of 100 μg/mL, the drug was then diluted with 100 mL of methanol and made up to the volume. The resultant solution was sonicated for about 2–3 min in order to dissolve the drug completely. 1 mL of the freshly prepared standard stock solution was pipetted and again diluted with 10 mL of methanol to give the concentration of 10 μg/mL. From the above working standard solution, a range of concentrations of 1–5 μg/mL solutions were made and scanned in a UV–visible double-beam spectrophotometer against methanol as a respective blank. At 250 nm wavelength, the absorbance was measured, and the linearity curve was plotted.^14^

#### 2.2.2 Solubility Studies

The drugs having inadequate aqueous solubility in oils, surfactants, and co-surfactants are the key criterion for the selection of the components for the development of SEDDS. Since the goal of this research work is to craft an orally administered SNEDDS formulation, it is crucial to consider the influence of drug solubility in the oil/lipidic phase on the potential of SNEDDS to effectively retain the solubilized drug state.^16^ In order to investigate solubility properties, an excessive amount of TCG drug was added to various types of oils, surfactants, and co-surfactants and mixed for about 5–10 min by using a vortex mixer. These mixtures were kept at 25 °C for 72 h in a shaking incubator. These samples were then centrifuged at 1500 rpm for 15 min for the removal of the insoluble drug. Methanol was used for diluting an aliquot of supernatant and was observed at 250 nm wavelength using a UV–visible double-beam spectrophotometer.

#### 2.2.3 Surfactant and Co-Surfactant Selection

The surfactant selection focused on the drug-solubilizing potential and the emulsification of the specified oily phase. The screening of co-surfactants was based on both their drug-solubilizing potential and their efficacy in increasing the selected surfactant nanoemulsification ability. The “hydrophilic–lipophilic balance” (HLB) was also used for choosing appropriate surfactant and co-surfactant for the purpose of developing SEDDS/SMEDDS/SNEDDS. Nonionic surfactants and co-surfactants are composed of a hydrophilic head (water-soluble group) and a hydrophobic tail consisting of fatty acids or fatty alcohols. The term used to describe the proportion of oil and water-soluble components is referred to as hydrophilic–lipophilic balance (HLB).^17^ Every surfactant and co-surfactant has a different HLB value according to the HLB system, and the selection of the oil phase for emulsion formation is based on a certain intended HLB number. By using surfactants and co-surfactants that possess the appropriate hydrophilic–lipophilic balance (HLB) value, the need for extensive trial and error procedures may be reduced, leading to the achievement of the most favorable formulation. Surfactants and co-surfactants within the HLB range of 8–18 exhibit optimal performance when used in oil-in-water formulations, and the HLB range of 4–6 is needed for water-in-oil emulsions. The suitable surfactant and co-surfactants having an optimum HLB number were selected to design an oil-in-water emulsion for the oil phase in which the TCG drug was highly soluble.^18^

#### 2.2.4 Pseudo-Ternary Phase Diagram

The suitable components for SNEDDS preparation were derived from the solubility results for the purpose of constructing the pseudo-ternary phase diagram. Clove oil as an oil phase, Tween-80 as a surfactant, and PEG-400 as a co-surfactant were utilized for the pseudo-ternary phase diagram construction. The water titration method was employed at ambient temperature for the identification of self-emulsification regions and for the selection of the appropriate concentrations of the Clove oil, Tween-80, and PEG-400 for the formulation of optimum SNEDDS. Surfactant and co-surfactant (S-mix) were combined in weighed ratios of (1:1), (2:1), and (3:1) in each group. Then, the oil and particular S-mix ratios were meticulously combined in varying ratios of (9:1, 8:2, 7:3, 6:4, 5:5, 4:6, 3:7, 2:8, and 1:9), respectively.^15,19^ The (oil:S-mix) mixtures were subjected to a titration method by gradually adding a fixed amount of water while stirring on a magnetic stirrer under mild continuous stirring. The combinations were visually examined for phase purity and flowability after the addition of water. The diluted mixtures were classified as turbid or transparent. The transparent and isotropic mixtures were identified as the microemulsion/nanoemulsion regions. Chemix School 11.0 software was used to plot the pseudo-ternary phase diagrams. The shaded region in a triangle plot was anticipated to be visually transparent, with one apex indicating the oil, the second one indicating water, and the third representing S-mix at a fixed weight ratio.^20^

#### 2.2.5 Formulation of SNEDDS of Ticagrelor

For the purpose of formulating a self-nanoemulsifying drug delivery system (SNEDDS) of TCG, various concentrations of oil, surfactant, and co-surfactant were selected based on pseudo-ternary phase diagrams. For creating a variety of SNEDDS formulations, different ratios of the selected excipients Clove oil, Tween-80, and PEG-400 were included in the experiment. The surfactant to the co-surfactant mixture (S-mix) was separately prepared by dissolving the required amounts of surfactant (Tween-80) and co-surfactant (PEG-400). The active drug ticagrelor (90 mg) was added to the clove oil in small portions under continuous stirring on a vortex mixer (VELP Scientifica, China). The S-mix was added to the oily phase containing the drug and mixed on a vortex mixer again for about 10–15 min. The formulations were then sonicated for 5–10 min to ensure the formation of a homogeneous mixture. The formulations were transferred to the orbital shaker (Biobase SK-0180-F, China) and kept under continuous shaking at 200 rpm for 72 h at room temperature. Finally, the obtained formulations were monitored visually for 48 h to observe any turbidity, phase separation, or precipitation that may occur prior to undertaking further assessment.^21^

### 2.3 Evaluation of Ticagrelor SNEDDS Formulations

#### 2.3.1 ζ Potential and ζ Size

The TCG-SNEDDS formulations (F1–F6) underwent a dilution process with distilled water at the ratio (1:100) stirred on the vortex mixer for 1 min and set aside for about 1 h. A Zetasizer (ZS 90 Malvern, U.K.) was utilized for the measurement of the ζ potential, polydispersity index (PDI), and ζ size. The ζ potential (mV) of the diluted solution of prepared TCG-SNEDDS was analyzed by utilizing a ζ dip cell. The ζ size (*d*, nm.) of the TCG-SNEDDS formulation was determined by the placement of the diluted SNEDDS solution onto the disposable sizing cuvettes at 25 °C.

#### 2.3.2 Self-Emulsification (SE) Time

Self-emulsification (SE) time is defined as the duration it takes the preconcentrate to transform into a homogeneous mixture when it is diluted. The TCG-SNEDDS (F1–F6) was added in a dropwise manner to 100 mL of the distilled water in a beaker and continuously stirred at 100 rpm on a magnetic stirrer. The time required for self-emulsification was visually observed and recorded. The emulsions are considered good if the emulsification time is less than 1 min having a clear bluish or transparent appearance, and bad if the emulsion becomes turbid.^22^

#### 2.3.3 Dispersibility test

In order to assess the capacity of SNEDDS to distribute evenly inside an emulsion and ascertain the dimensions of the resulting globules, a dispersibility test is performed. 0.1 mL of the prepared TCG-SNEDDS (F1–F6) was introduced into 250 mL of distilled water and stirred using a magnetic stirrer at 100 rpm, and the duration required for the emulsion development was documented. The SNEDDS formulation creates a variety of mixtures upon dilution with distilled water according to which the *in vitro* activity may be graded.^23^ Grading system is given in Table 1.

**Table 1:** Emulsion Grading System for the Assessment of the Emulsion Formed upon Dilution

emulsion grade	appearance of the emulsion formed	time taken
A	rapidly forming having a transparent appearance	<1 min
B	rapidly forming, somewhat less clear with a bluish or whitish appearance	<1 min
C	cloudy or milky appearance	about 2 min
D	dull, grayish, or little white slow emulsifying with a mild oily appearance	>2 min
E	poorly emulsified formulations with big oil globules	>2 min

#### 2.3.4 Phase Separation and Stability Test

A volume of 1 mL of prepared TCG-SNEDDS formulations was combined with 10 mL of distilled water, phosphate buffer solution 6.8 pH (PBS 6.8), as well as 0.1 N HCl at 37 °C, stirred on a magnetic stirrer for some time, and then kept aside. After 24 h, the diluted formulations were assessed visually for any kind of phase separation or precipitation, exhibiting that all of the formulations remained stable upon dilution.^24^

#### 2.3.5 Thermodynamic Stability Studies

The physical stability parameter of TCG-SNEDDS formulations is essential for their therapeutic efficacy since drug precipitation in an excipient matrix is a possibility. Excipient phase separation can be driven by poor formulation, and its physical stability might affect the bioavailability of excipients and therapeutic effectiveness. Additionally, it is important to consider that the gelatin shell of the capsules may exhibit incompatibility with the formulation, potentially resulting in undesirable characteristics, such as fragility, softness, prolonged disintegration, or inadequate drug release. The investigations include the following cycles:

##### 2.3.5.1 Freeze–Thaw Stress Cycle

The TCG-SNEDDS formulations and distilled water were mixed thoroughly in a ratio of 1:10, and then these diluted formulations were put through the three cycles between −21 and 25 °C to resume the initial state of the formulation, with at least 48 h of keeping at each temperature. Formulations that passed this test exhibited great stability without any physical change, such as precipitation, creaming, or cracking.

##### 2.3.5.2 Heating and Cooling Cycle

The TCG-SNEDDS formulations and distilled water are mixed in a ratio of 1:50 and subjected to six cooling and heating cycles between the lower temperature, 4 °C, and the higher temperature, 45 °C, with a maximum exposure time of at least 48 h. The formulations that successfully passed the heating and cooling tests were then subsequently subjected to a centrifugation test.

#### 2.3.6 pH

The pH of the TCG-SNEDDS formulations was measured by using a digital pH meter (Adwa AD1030, Romania).

#### 2.3.7 Cloud Point Determination

Cloud point is the temperature at which the clear formulation turns cloudy.^25^ The TCG-SNEDDS formulations underwent a dilution process utilizing the distilled water in a ratio (1:100) and placed in a beaker inside the water bath at 37 °C. The temperature of the water bath was incrementally raised by 1 °C at a time. The diluted formulation was cooled, and this procedure was performed to ensure reliability. Moreover, the temperature was gradually increased, and the cloud point was assessed.

#### 2.3.8 X-ray Diffraction Analysis (XRD)

In this test, pure TCG drug, TCG-SNEDDS (F4), and TCG-SNEDDS (F6) were analyzed by X-ray diffraction (Malvern Instruments, U.K.) utilizing Ni-filtered Cu Kα radiations. At room temperature, the samples were subjected to examination at an angular position of 2θ, ranging from 5 to 70°, while the XRD patterns were being recorded. The step size for the continuous scan mode was set at 0.02°/s. To determine whether or not the formation of TCG-SNEDDS has altered the crystal structure of TCG, changes in the location of diffraction peaks of TCG were studied.^26^

#### 2.3.9 Fourier Transform Infrared (FTIR) Spectroscopy

FTIR spectroscopy of TCG pure drug, Clove oil, Tween-80, PEG-400, TCG-SEDDS (F4), and TCG-SEDDS (F6) was conducted using a digital FTIR spectrometer (Thermo Fischer Scientific) covering a spectral range of 650–4000 cm^–1^. The spectrophotometer was outfitted with a diamond ATR interferometer (attenuated total reflectance), which had an adjustable speed range of 0.1–4 cm/s. The diameter of the infrared (IR) beam used in the investigation varied between 2 and 11 mm. The FTIR spectra were obtained at a spectral resolution of 4 and were replicated several times (8 times) in order to facilitate molecular analysis and gather insights into the chemical structure. To ensure precise results, correct outcomes were produced by calculating the average values of eight scans for all samples.^26^

#### 2.3.10 Scanning Electron Microscopy (SEM) Analysis

Structural morphology analysis of TCG pure drug, TCG-SNEDDS (F4), and TCG-SNEDDS (F6) formulations was observed by scanning electron microscopy, SEM (ZEISS). A certain amount of sample was deposited in vacuum on a platinum SEM stub, which featured a very thin coating layer onto the silicon chip and was observed at an accelerating voltage of 20 kV. The structures and configurations of the pure TCG drug, TCG-SNEDDS (F4), and TCG-SNEDDS (F6) formulation particles were photographed. SEM is a method of constructive characterization that provides details about the topography (particle surface properties) and morphology (particle size, shape, and organization) of the samples being analyzed. Thermionic guns are used in SEM as an electron source in addition to other parts.^27,28^

#### 2.3.11 Differential Scanning Calorimetry (DSC) Analysis

The fluctuations in the energy of TCG (Pure drug), TCG-SNEDDS (F4), and TCG-SNEDDS (F6) were investigated by a method of thermal analysis by utilizing a thermal analyzer instrument, differential scanning calorimetry (SDT Q600 V8.3 Build 101). A thermoanalytical technique is used to quantify the changes in thermal energy required for elevating the temperature of the sample relative to a reference material. The experiment included placing a limited quantity of samples (ranging from 1 to 78 mg) into individual aluminum pans, which were then subjected to heating within a temperature range of 25 to 500 °C. DSC thermograms were obtained using a 10 °C/min heating rate and a 1.5 W/g DSC heat flow.^29^

#### 2.3.12 Thermogravimetric Analysis (TGA)

In the thermogravimetric analysis (TGA), samples consisting of approximately 10 mg each of pure TCG drug, TCG-SNEDDS formulations F4 and F6, were carefully deposited onto the aluminum pans. These pans were then subjected to a gradual temperature incremeting changes in the weight were utilized to determine the temperature at which specific weight loss percentages occur.^30^

#### 2.3.13 Entrapment Efficiency

The centrifugation method, an indirect technique, was used to determine the percentage entrapment efficiency. For this, the optimized TCG-SNEDDS formulations F4 and F6 were centrifuged at 10,000 rpm for 45 min in a centrifuge. 1 mL of the supernatant was dissolved in 10 mL of methanol. The amount of the TCG free/unbound drug entrapped in the supernatant was measured through UV spectroscopy at 250 nm wavelength. Each sample was analyzed three times. The aforementioned calculation was used to obtain the percentage of entrapment efficiency.
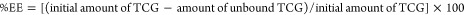


#### 2.3.14 *In Vitro* Drug Release Study

The *in vitro* drug release from the pure TCG suspension and TCG-SNEDDS formulations F4 and F6 was evaluated by utilizing a USP dissolution apparatus Type II with a rotating speed of 100 rpm at 37 °C. The dialysis bag method was used for this investigation to prevent the intervention of the unreleased drug by ensuring that only unbound drugs make it to dissolution media. The dialysis membranes were submerged for a day in the dissolution media, *i.e.*, 0.1 N HCl and PBS 6.8, at ambient temperature. 90 mg of TCG-SNEDDS formulations underwent dilution with 10 mL of distilled water, and 5 mL of diluted formulations and pure drug suspensions were filled in presoaked dialysis membranes. The dialysis membranes were fastened to the rotating paddles and submerged in the dissolution mediums after being firmly tied at both ends to avoid leaking. An aliquot of 5 mL of the sample was pipetted out of the dissolution vessel at regular intervals of 0.25, 0.5, 1, 1.5, 2, 3, 4, 5, 6, and 12 h. To maintain the sink condition, 5 mL of new dissolving medium was added. A UV spectrophotometer was used to examine the samples for drug release at 250 nm wavelength.^31^

#### 2.3.15 *In Vitro* Kinetic Drug Release Modeling

To evaluate the drug release behavior and kinetics of TCG-SNEDDS, a variety of *in vitro* kinetic models, including zero and first order as well as Korsmeyer–Peppas, were applied to the collected data.^29^

#### 2.3.16 *Ex Vivo* Drug Permeation Study

A noneverted gut sac approach was used to examine the *ex vivo* permeation of TCG drug from its pure suspension form and the chosen TCG-SNEDDS formulation F4. The testing protocol approval was received from the Ethical Review Committee (ERC) of the University of Central Punjab. For this study, a male albino rabbit weighing about 1–1.3 kg was sacrificed, and the small intestine was removed from its body. 5–6 cm of jejunum was cut off and rinsed with Krebs ringer phosphate buffer and normal saline at 37 °C. A 3 mL syringe was used to fill the jejunum sac with 1 mL of pure TCG suspension and TCG-SEDDS formulation F4 and tied at both ends with a cotton thread. The intestines were suspended into 250 mL of PBS 6.8 containing 0.2% of polysorbate stirred at 50 rpm maintaining the temperature at 37 °C. At the time intervals of 0.5, 1, 1.5, 2, 2.5, and 3 h, 3 mL of the sample was withdrawn from the beaker and replenished with the same amount of PBS 6.8. The absorbance of the samples was analyzed by UV spectrophotometer at a wavelength of 250 nm, and calculations were carried out regarding the apparent permeability and permeation flux by applying the values to the below equation^32^
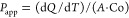


#### 2.3.17 Pharmacodynamic Study

The pharmacodynamic study was conducted to assess and compare TCG-SNEDDS formulation F4 and pure TCG drug suspension, administering an oral dosage of 8 mg/kg. This test procedure approval was received from the Ethical Review Committee (ERC) of the University of Central Punjab. A total of 6 albino male rabbits weighing between 1 and 1.5 kg each were included in this study. The rabbits were housed under controlled laboratory conditions at 25 ± 2 °C and 50 ± 5% (RH). Prior to the commencement of treatment, all rabbits were subjected to marking, and a blood sample of 0.5–1 mL was extracted from the lower limb. Three groups of albino rabbits were made: group 1 received pure TCG dose (8 mg/kg), group 2 received TCG-SNEDDS F4 (8 mg/kg) at an equivalent dose, and group 3 was considered the control group. The administration of the dosage was conducted through oral gavage, and subsequent blood samples were obtained at 24 h intervals. These samples were collected by using anticlotting tubes that contained heparin. Blood samples underwent analysis for complete blood count (CBC) and erythrocyte sedimentation rate (ESR). Hematology analyzer SYSME KX-21 was used to perform tests.^28,33^

#### 2.3.18 Statistical Analysis

GraphPad Prism version 9.2 was used to conduct statistical analysis. One-way ANOVA (analysis of variance) was performed with a 95% confidence interval following Tukey’s multiple compression test. This analysis was applied to *in vitro* drug release data to discern the distinction between TCG-SNEDDS formulations and the pure TCG suspension. To assess the significance of differences in *ex vivo* permeation data, Student’s *t* test was employed, and statistical significance was defined as *p*-value < 0.05.^29^

## 3 Results and Discussion

### 3.1 Linearity Curve Determination of Ticagrelor

**Figure 1:**
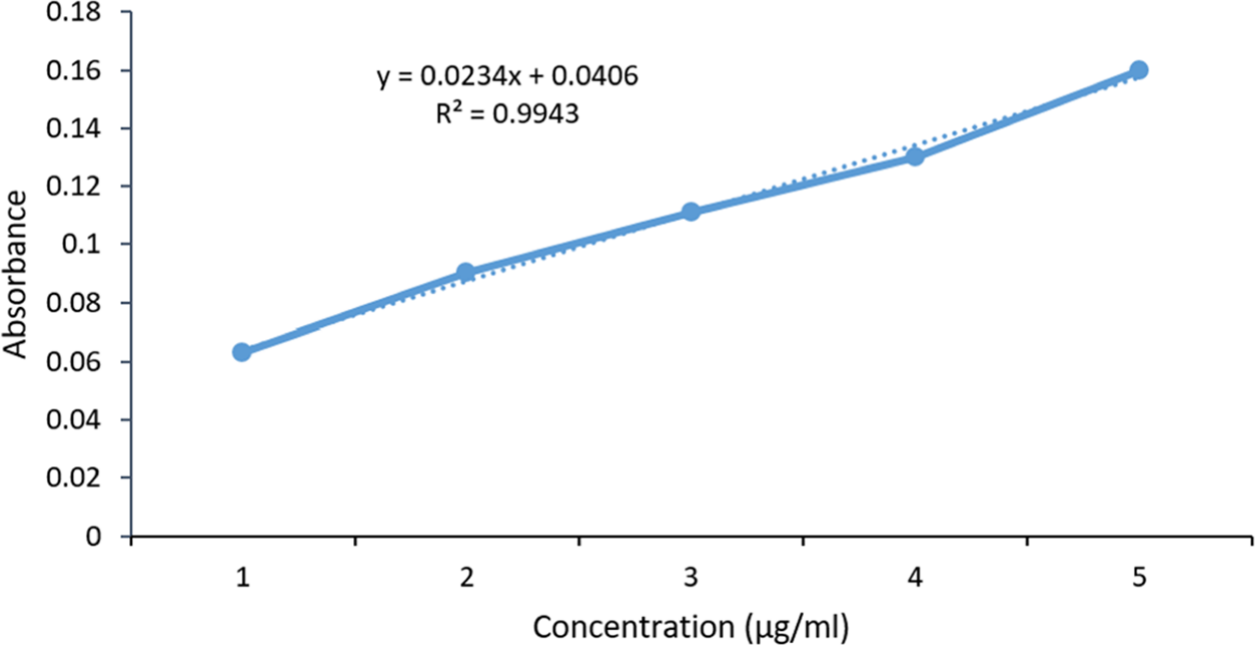
TCG linearity curve in methanol.

The linearity curve for TCG in methanol was established with concentrations ranging from 1 to 5 μg/mL. The linear regression equation was determined to be (*y* = 0.0234*x* + 0.0406), and it had a high correlation coefficient (*R*^2^) of 0.9943. The linearity curve is depicted in Figure 1.

### 3.2 Solubility Studies

**Figure 2:**
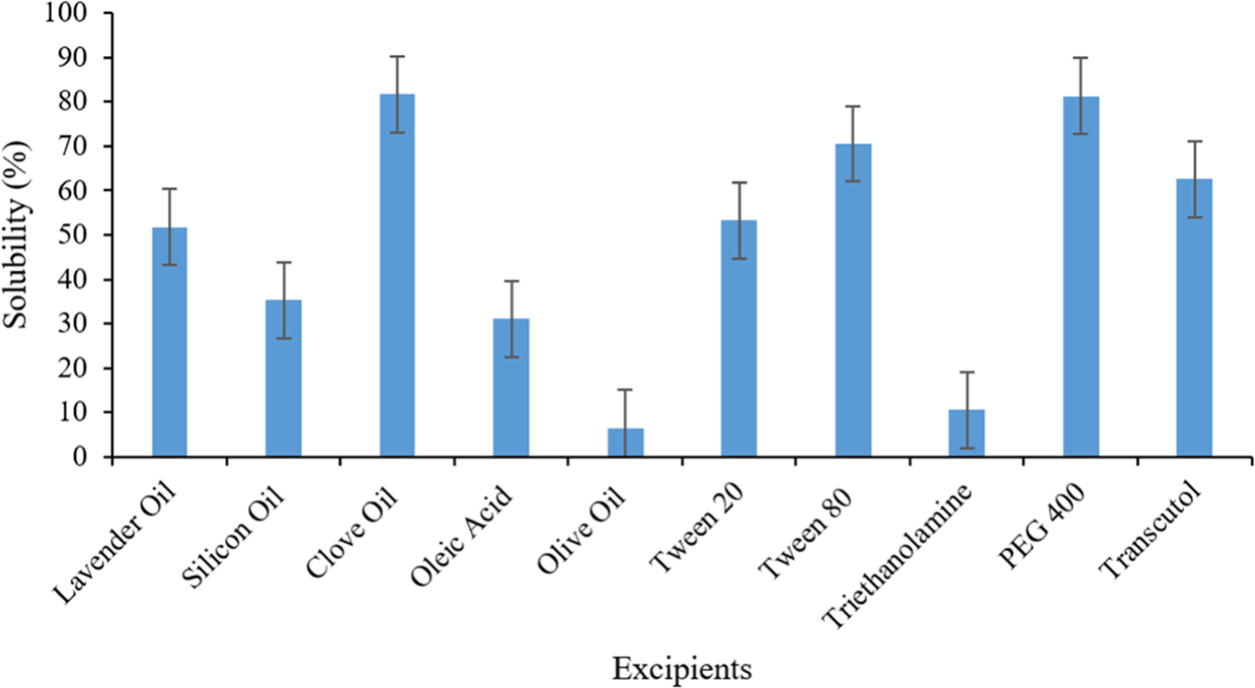
Solubility profile of TCG in various oils, surfactants, and co-surfactants.

The excipients to be utilized in the formation of SNEDDS formulations must exhibit higher solubility for the drug to provide optimum drug solubilization and to avoid drug precipitation in the gut lumen. Figure 2 presents the solubility data pertaining to TCG in various oils, surfactants, and co-surfactants. Clove oil, Tween-80, and PEG-400 were found to have a maximum solubility of TCG, respectively.

### 3.3 Surfactant and Co-Surfactant Selection

The optimum surfactant and co-surfactant for clove oil must have an HLB value of about 13.93. Tween-80 and PEG-400 appear to be the most suitable choice to formulate emulsion with clove oil since they have HLB values of 15 and 13, respectively. Therefore, Tween-80 and PEG-400 were identified as optimal surfactants and co-surfactants for the incorporation of TCG in SNEDDS formulations having clove oil as an oily phase.^34^

### 3.4 Pseudo-Ternary Phase Diagram

Pseudo-ternary phase diagrams were constructed in order to determine the self-nanoemulsifying zone and choose optimal ratios of oil and S-mix (surfactant and co-surfactant mixture) for the development of self-nanoemulsifying DDS. These ternary phase diagrams are crucial for understanding nanoemulsion phase behavior and for the optimization of the SEDDS. The ternary diagram consists of three components: oil, water, and S-mix. Each corner of the figure represents one of the components with a 100% concentration. In this experimental study, the surfactant used was Tween-80, the co-surfactant utilized was PEG-400, and the oily phase consisted of clove oil. The surfactant-to-co-surfactant (S-mix) ratios used in this study were (1:1), (2:1), and (3:1). The different ratios of S-mix were incorporated into the different ratios of selected oil, and the resultant combinations were titrated with a fixed amount of water.^35^

**Figure 3:**
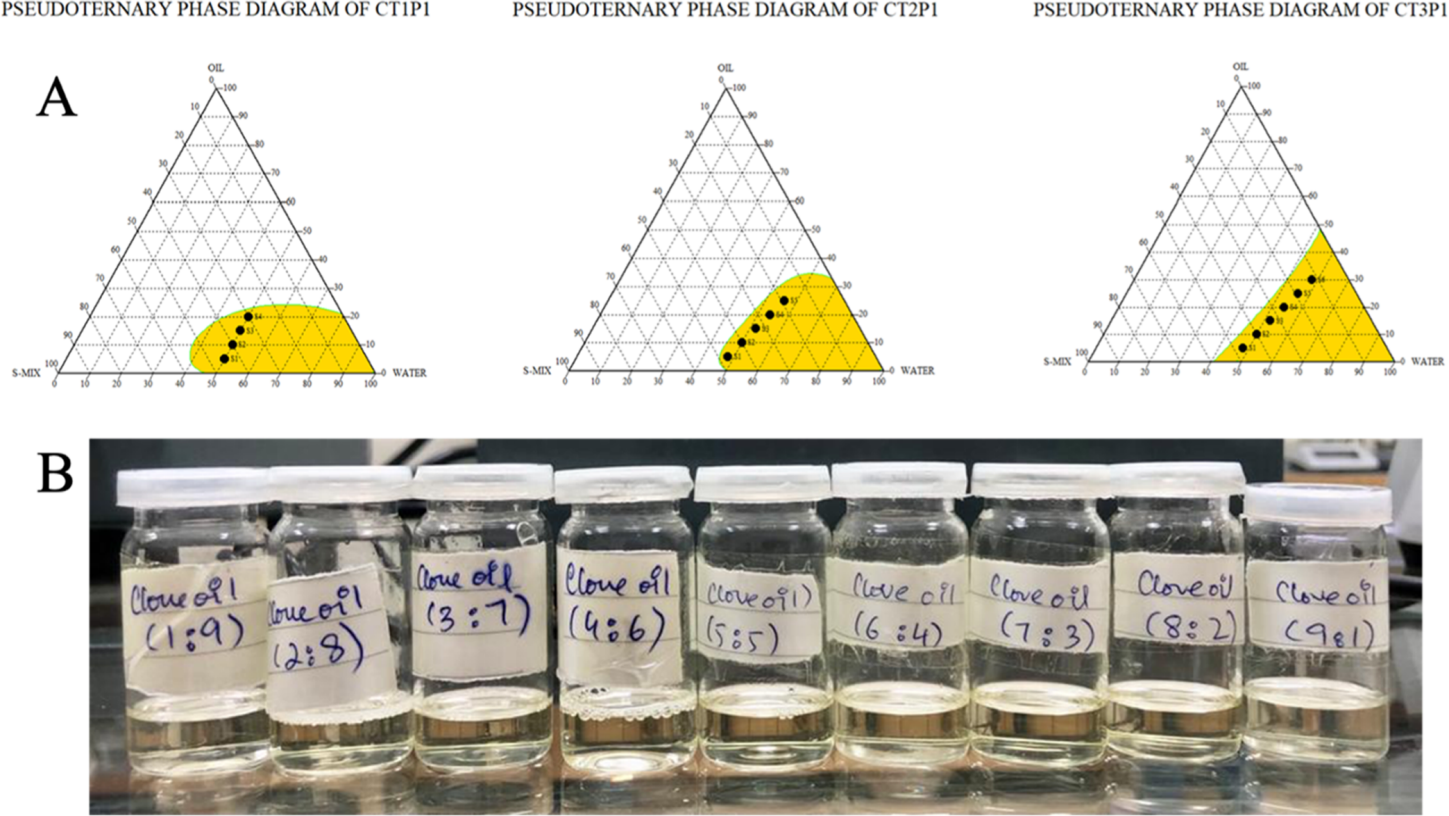
Pseudo-ternary phase diagrams of CT1P1, CT2P1, and CT3P1 showing yellow-colored nanoemulsion region (A) and optically transparent emulsions formation upon water titration for pseudo-ternary phase diagram plot (B).

The introduction of PEG-400 into the SE region was found to enhance the self-emulsification process spontaneity. The emulsification efficiency was notably favorable when the S-mix concentration exceeded 75% in the SNEDDS formulation. Conversely, it was observed that spontaneous emulsion formation proved to be ineffective when the surfactant content in the SNEDDS was below 50%. After the aqueous titration, these results were incorporated into the Chemix software for constructing pseudo-ternary phase diagrams. Figure 3a illustrates the several regions of the triangles (CT1P1, CT2P1, and CT3P1), with the colored parts representing the nano area. This particular region is characterized by its transparency and monophasic nature, as shown in Figure 3b. The best self-nanoemulsifying activity and ideal intermolecular contact between oil, S-mix, and water indicate a broad nanoemulsion region. Figure 3a also demonstrates that an increase in the surfactant-to-co-surfactant ratio leads to a bigger nano region. However, as the ratio of oil was increased, the streaks of oil were visible upon the water titration.^36^

### 3.5 ζ Potential, ζ Average, and PDI

**Figure 4:**
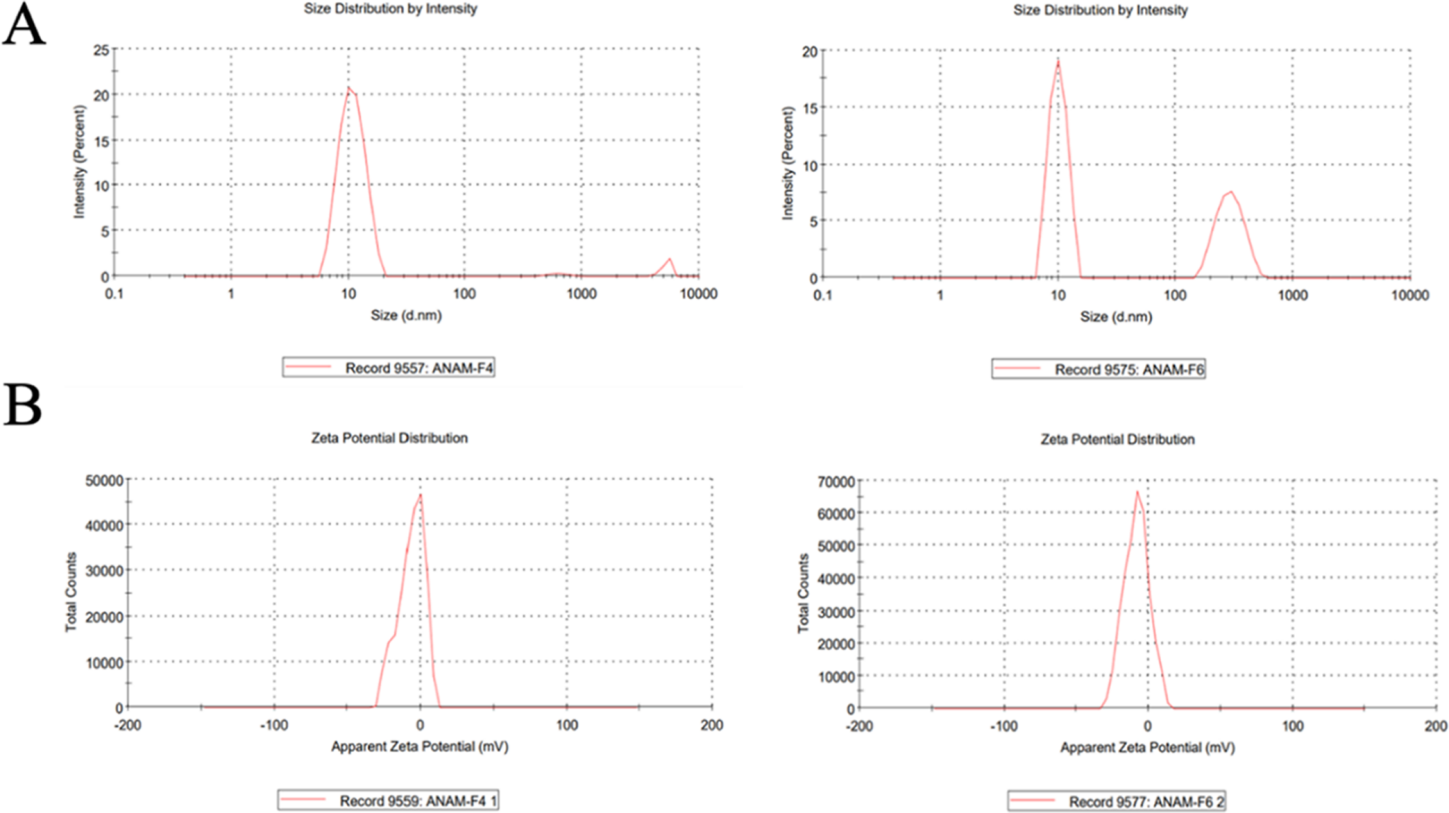
ζ size (A) and ζ potential (B) of TCG-SNEDDS F4 and F6

The ζ potential, polydispersity index (PDI), and ζ average of TCG-SNEDDS formulations were assessed. The ζ size (*d*, nm) was found to be in the range of 11.85–206.4 nm and the PDI of all of the TCG formulations was below 0.5, indicating uniform distribution of particle size. The ζ potential was determined to be in the −9.92 to −6.23 mV range. The value of the ζ potential plays a pivotal role in determining the stability of colloidal dispersions. Typically, a colloidal dispersion is considered stable when its ζ potential falls within the range of −10 to +10 mV. Values exceeding +30 mV or less than −30 mV are indicative of a strongly cationic and strongly anionic, respectively.^37^ It was determined from the data that TCG formulations F4 and F6 exhibited less ζ size in comparison to all other formulations. The PDI of all TCG formulations was below 0.5 as shown in Table 2 and Figure 4a,b.

**Table 2:** List of the ζ Potential (mV), ζ Size (*d*, nm), and PDI of TCG-SNEDDS

formulation	ζ potential (mV)	ζ size (*d*, nm)	PDI
F1	–7.65	150.1	0.360
F2	–6.30	206.4	0.467
F3	–8.96	157.3	0.465
F4	–6.35	11.85	0.274
F5	–6.23	94.53	0.239
F6	–8.21	77.17	0.368

### 3.6 Self-Emulsification (SE) Time

TCG-SNEDDS formulations were assessed for self-emulsification (SE) time according to visual inspection. Self-emulsifying combinations should dissolve easily in water while being gently shaken. Table 3 enlists SE times that were established for the prepared TCG-SNEDDS formulations. All formulations were found to emulsify in less than 1 min indicating good performance in all formulations.

**Table 3:** Self-Emulsification Time (sec) of TCG-SNEDDS (F1–F6)

formulations	self-emulsification time (s)	results
F1	18	good
F2	19	good
F3	18	good
F4	18	good
F5	20	good
F6	20	good

### 3.7 Dispersibility Test

All of the TCG-SNEDDS formulations were found to be clear within 1 min after performing the dispersibility test. They are highly apparent and of grade-A quality.

### 3.8 Phase Separation and Stability Test

The TCG-SNEDDS formulations underwent dilution using water, PBS 6.8, and 0.1 N HCl. The diluted samples were then incubated for a duration of 24 h, during which the occurrence of drug precipitation or phase separation was visually assessed. All formulations were resistant to dilution since no indication of phase separation or precipitation was seen. Table 4 presents the findings.

**Table 4:** Phase Separation and Stability Test Results

formulations	water	0.1 N HCl	PBS 6.8
F1	√	√	√
F2	√	√	√
F3	√	√	√
F4	√	√	√
F5	√	√	√
F6	√	√	√

### 3.9 Thermodynamic Stability Studies and pH

The primary aim of the thermodynamic stability research is to identify formulations that exhibit metastability. During the freeze–thaw cycle, heating–cooling cycle, and centrifugation at 6000 rpm for 15 min, the emulsions remained stable, indicating no phase separation or precipitation. The results are listed in Table 5 along with the pH of all formulations (F1–F6).

**Table 5:** Thermodynamic Results of TCG-SNEDDS (F1–F6)

formulations	freeze–thaw cycle (−20 and 25 °C)	heating and cooling cycle (4 and 45 °C)	centrifugation (6000 rpm for 15 min)	pH
F1	√	√	√	6.6
F2	√	√	√	6.4
F3	√	√	√	6.8
F4	√	√	√	6.7
F5	√	√	√	6.5
F6	√	√	√	6.6

### 3.10 Cloud Point Determination

Cloud of the prepared TCG-SNEDDS formulations was observed to exceed 60 °C, indicating that the nanoemulsion region would maintain its stability at physiological temperatures, hence eliminating the possibility of phase separation or precipitation. Moreover, all TCG-SNEDDS formulations exhibited a cloudy appearance after 74.7 °C. It may be due to the precipitation of the drug.

### 3.11 X-ray Diffraction Analysis (XRD)

**Figure 5:**
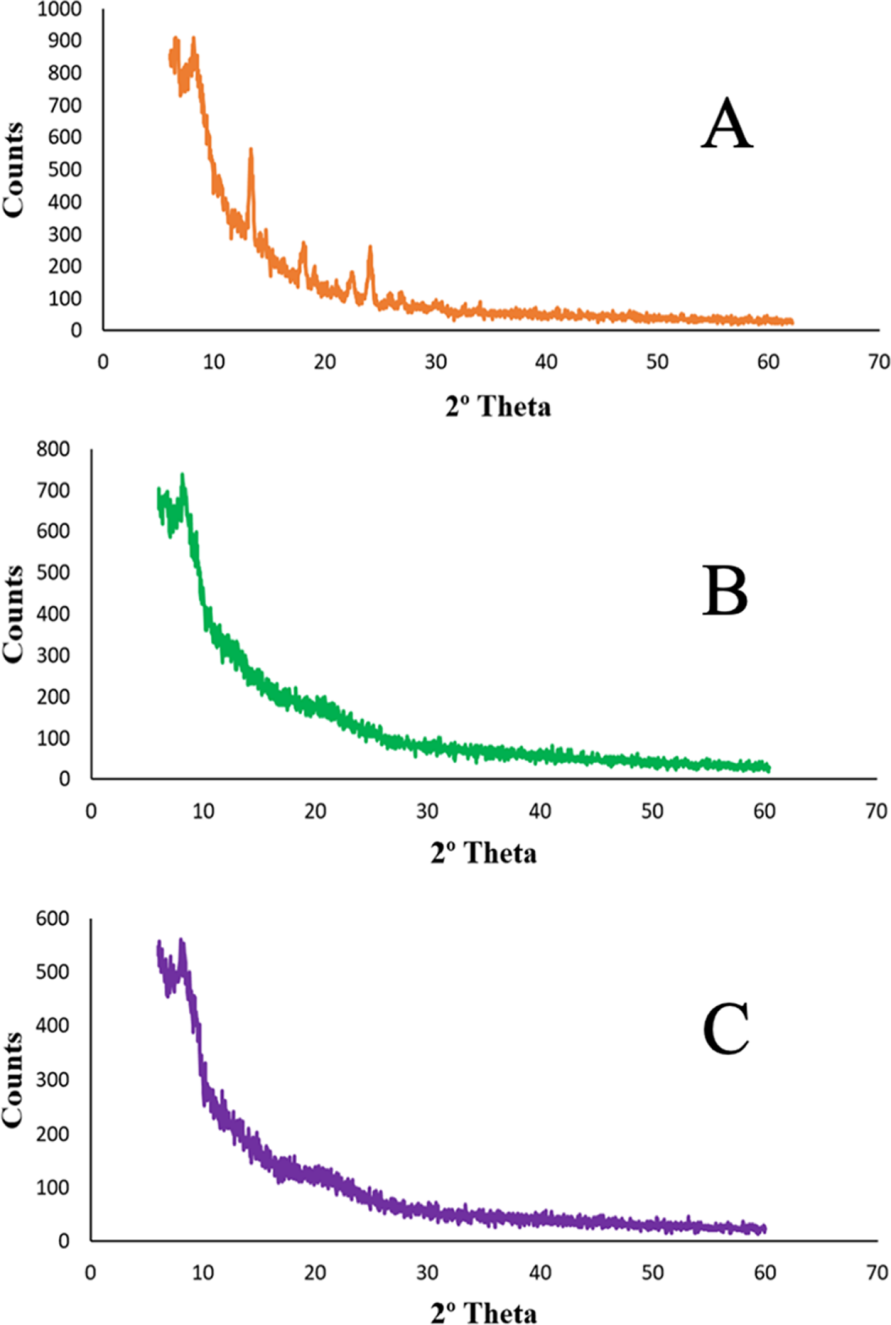
XRD spectra of (A) pure TCG, (B) TCG-SNEDDS F4, and (C) TCG-SNEDDS F6.

The crystalline structure of pure TCG was shown by the presence of many high-intensity peaks. The major high-intensity peak of pure TCG was seen at 13.08 °C, and also some minor peaks were observed at 18.22, 22.36, and 24.83 °C. In the XRD patterns of the TCG-SNEDDS formulations F4 and F6, no specific peaks were observed, indicating that the crystallinity of TCG was transformed into a solubilized or amorphous state. These outcomes demonstrated the successful entrapment of TCG in SNEDDS formulations as shown in Figure 5.^38^

### 3.12 Fourier Transform Infrared (FTIR) Spectroscopy

**Figure 6:**
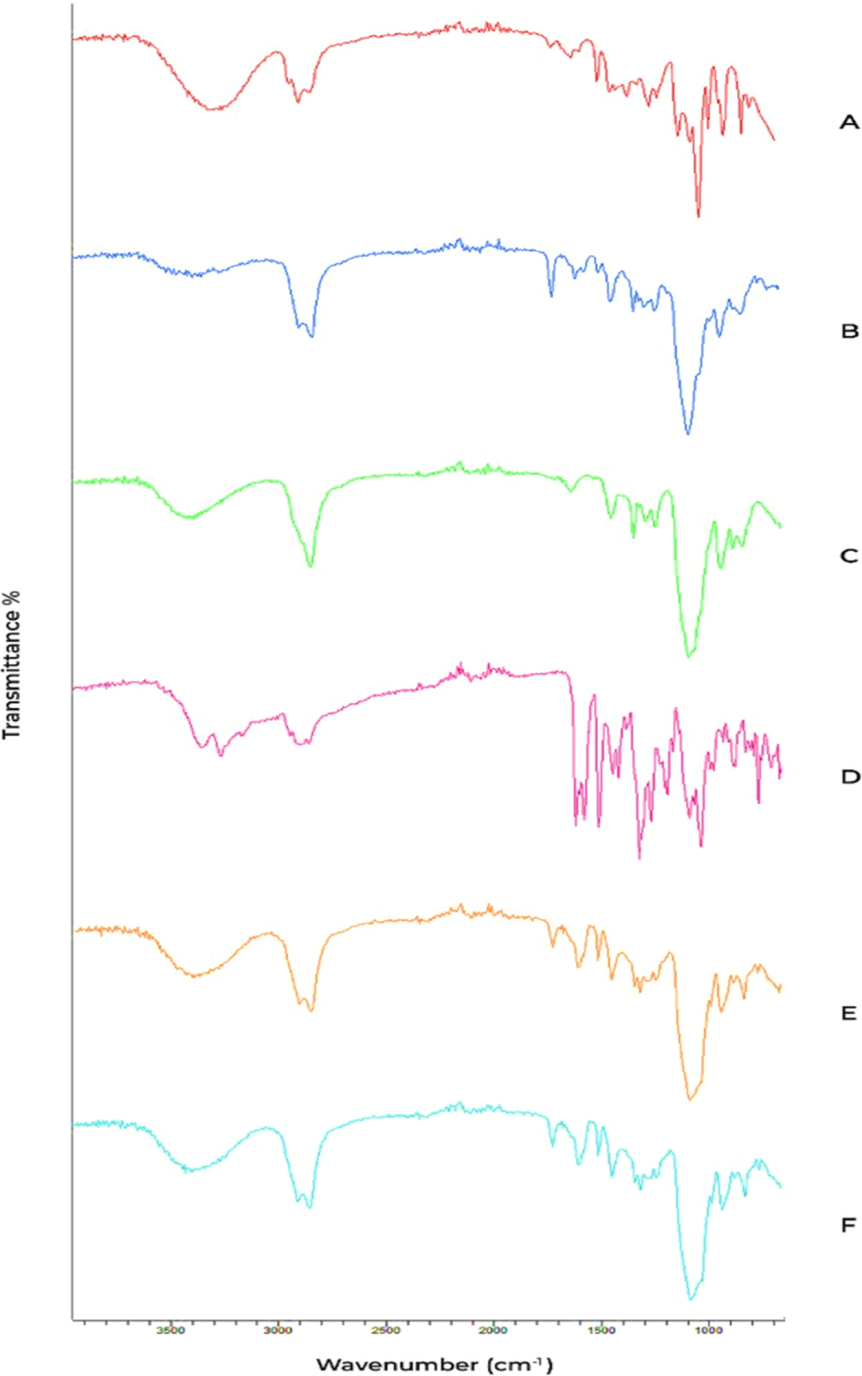
FTIR spectra of (A) Clove Oil, (B) Tween-80, (C) PEG-400, (D) Ticagrelor, (E) TCG-SNEDDS F4, and (F) TCG-SNEDDS F6.

The FTIR spectra of the pure TCG drug displayed distinct absorption peaks at several wavenumbers, as shown in Figure 6. These peaks were seen at 3403, 3288, 2933, 2854, 1605, 1558, 1505, 1455, 1316, 1274, 1256, 1210, 1110, 1091, and 761 cm^–1^. The appearance of −OH stretch and −NH stretches was suggested by absorption bands seen at 3288 and 3403 cm^–1^, respectively. Additionally, the absorption bands observed at 2933 and 2854 cm^–1^ were indicative of alkyl stretch (−CH). The existence of the −N–H stretch was indicated by the peaks seen at 1605 and 1558 cm^–1^. Similarly, the absorption band observed at 1455 cm^–1^ provided evidence of the existence of the methyl bend. Additionally, the peaks observed at 1256 and 1210 cm^–1^ were indicative of the −C–OH stretch. The existence of the −C–O stretch may be inferred from the distinct peaks seen at 1110 and 1091 cm^–1^.^39^

The OH group and aromatic eugenol structure, which is a significant component of the clove oil, were identified as the typical absorption bands of (O–H) phenolic and stretching of C–H in the aromatic ring seen at 3515 and 3076 cm^–1^, respectively. Because of the presence of the allyl group in eugenol (C–H attached with Olefin), the (C–H) stretching absorption band at 3003 cm^–1^ was noticed.^40^

PEG-400 and Tween-80 in the FTIR spectrum exhibited absorption bands at approximately 3600, 2900, 1070, 940, and 800 cm^–1^. The O–H, C–H (vibrations of the – CH2 group), and C–O stretching displayed absorption peaks at 3300, 2900, and 1245 cm^–1^. The C–H bending vibrations of the – CH2 group displayed a peak at 1452 cm^–1^, although asymmetrical bending vibrations of −CH3 were also present. C–O–C symmetrical stretching was attributed to the peak in close proximity of around 900 cm^–1^. Tween-80 showed a distinctive peak at 1735 cm^–1^ linked to the C–O group. The absorption bands seen in the spectra of TCG-SNEDDS F4 and F6 were found to be in regions comparable to those of the pure TCG drug. Furthermore, no interaction was observed among the prominent peaks. TCG drug demonstrates chemical stability when included inside of the TCG-SNEDDS formulations F4 and F6. This phenomenon may be attributed to the similarity in functional linkages present in the surfactant, co-surfactant, and oil phases used. Consequently, the absorption bands seen are found to be located in comparable regions, as shown in Figure 6.^41^

### 3.13 Scanning Electron Microscopy (SEM)

**Figure 7:**
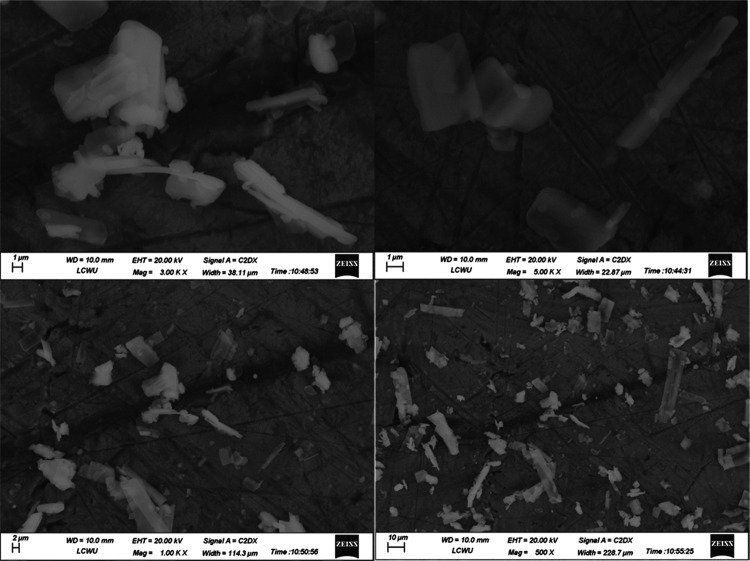
SEM images of pure TCG.

**Figure 8:**
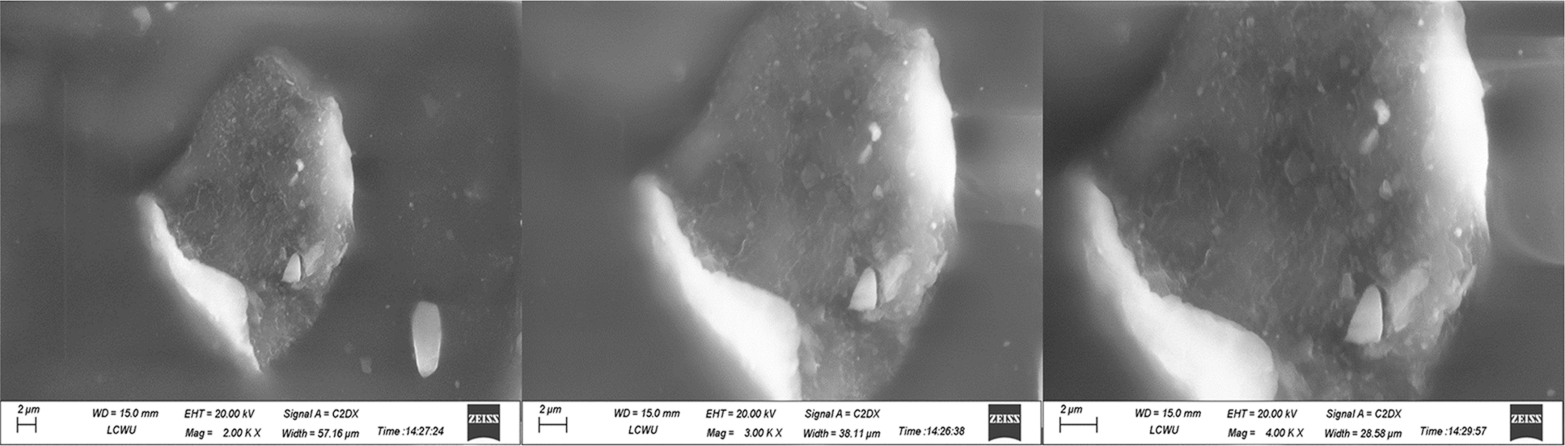
SEM images of TCG-SNEDDS F4.

**Figure 9:**
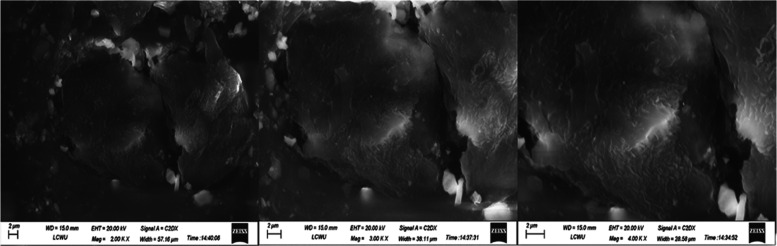
SEM Images of TCG-SNEDDS F6.

The scanning electron microscopy (SEM) images of TCG pure drug F4, and F6 are displayed in Figure 7, Figure 8, and Figure 9, respectively. The surface morphology of pure TCG exhibited oblong, rod-shaped crystalline structures having irregular morphology and uneven rough agglomerates, as seen in Figure 7. The SEM images of TCG-SNEDDS formulations F4 and F6 exhibited no rod-shaped crystalline morphology, thereby indicating the effective integration of TCG into the SNEDDS formulation, as seen in Figures 8 and 9, respectively.

### 3.14 Differential Scanning Calorimetry (DSC) Analysis

**Figure 10:**
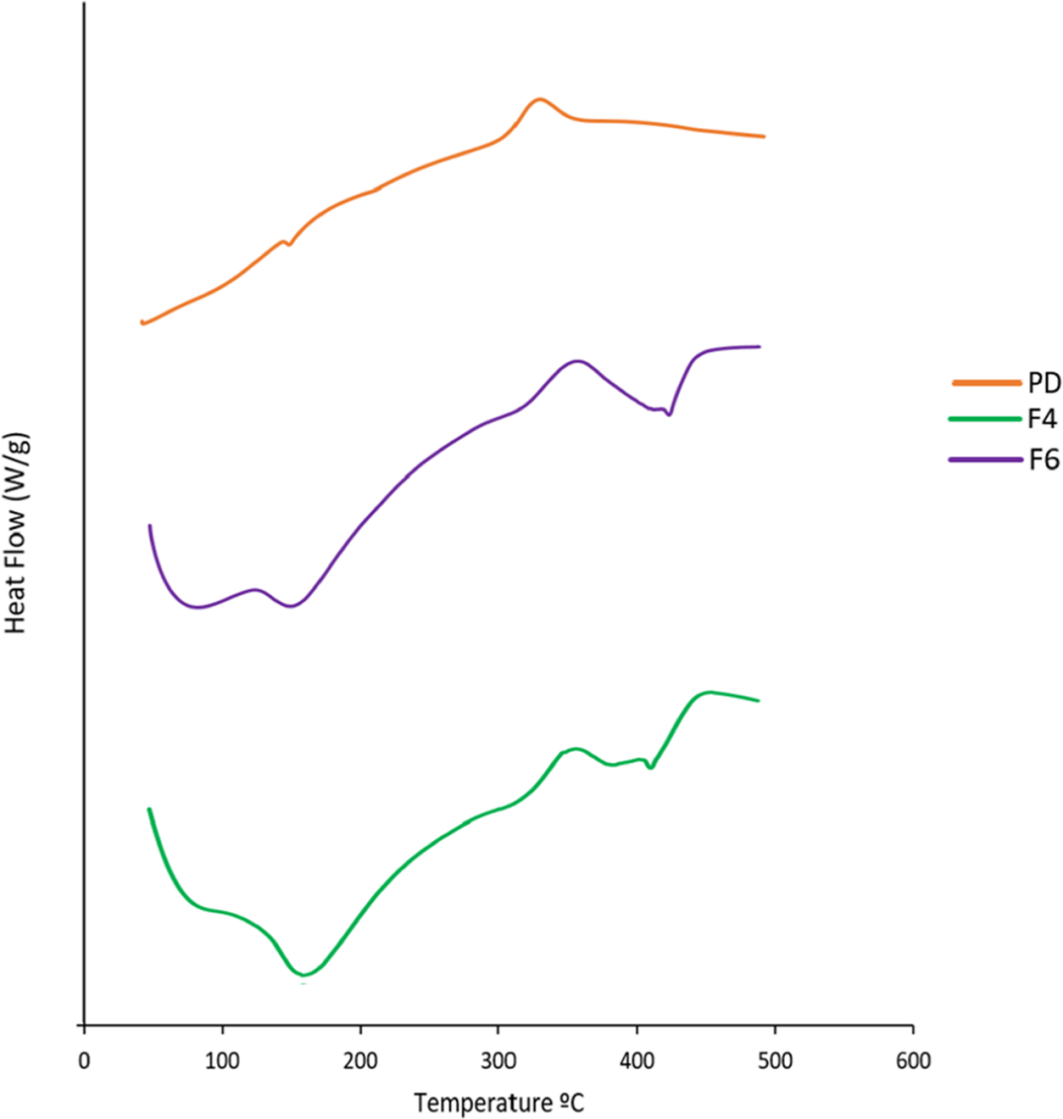
DSC curves of TCG pure drug (PD), TCG-SNEDDS F4 and F6.

DSC thermograms for TCG pure drug, TCG-SNEDDS F4 and F6, were obtained. The endothermic peak of pure TCG, which represented the melting point (MP) of TCG, was observed at 137.41 °C and an exothermic peak was seen at 337.5 °C. The TCG-SNEDDS formulations F4 and F6 demonstrated wider peaks, accompanied by an increase in melting points, specifically at 413 and 440 °C, respectively. The increase in the melting point of TCG-SNEDDS could be ascribed to the presence of additional constituents, such as surfactant and co-surfactant, which may interact with the TCG in a way that may alter its thermal behavior within the SNEDDS formulations. The endothermic peak of TCG was not seen in the DSC curves of TCG-SNEDDS F4 and F6 due to the amorphous form. The TCG-SNEDDS endothermic peaks at higher temperatures could be indicative of the energy required to disrupt the bonds between TCG and the excipients in the SNEDDS formulations. The observed phenomenon confirms the transition from the crystalline form of TCG into the amorphous form caused by the creation of the SNEDDS formulations. The primary aim of performing DSC was to check the anticipated molecularly dissolved state of TCG which signifies the transformation from a highly crystalline state to a less crystalline state due to the formation of SNEDDS as shown in Figure 10.^39^

### 3.15 Thermogravimetric Analysis (TGA)

**Figure 11:**
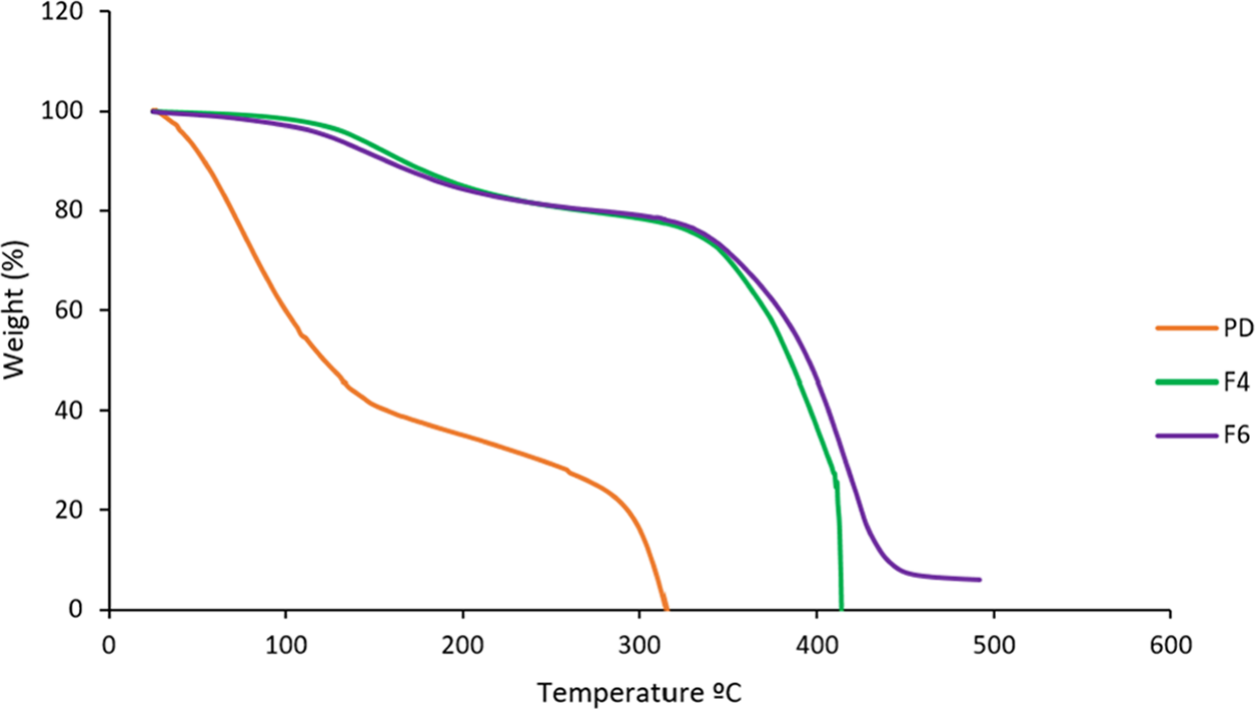
TGA curves of pure TCG drug (PD), TCG-SNEDDS F4 and F6.

TGA was employed to assess the thermal stability of both the prepared TCG-SNEDDS formulations F4 and F6 as well as pure TCG drug. TCG pure drug exhibited a swift reduction in weight, commencing at 24.51 °C and continuing until 314.86 °C, with nearly 50% of the drug decomposing at 121.31 °C. Beyond 314 °C, no further weight loss was observed as depicted in Figure 11. Conversely, TCG-SNEDDS formulation F4 displayed a gradual decrease in weight starting at 100 °C, with approximately 40% of the formulation decomposing at 368 °C. At 413 °C, the complete decomposition of TCG-SNEDDS F4 can be observed. Similarly, TCG-SNEDDS F6 exhibited a slow weight loss initiating from temperature 100 to 490 °C. However, the outcomes revealed that the TCG-SNEDDS formulations exhibited stability even at elevated temperatures in comparison to the pure TCG as shown in Figure 11.^42,43^

### 3.16 Entrapment Efficiency

The entrapment efficiency of TCG-SNEDDS F4 and F6 was calculated to be 98.93 and 97.70%, respectively.

### 3.17 *In Vitro* Drug Release Study

**Figure 12:**
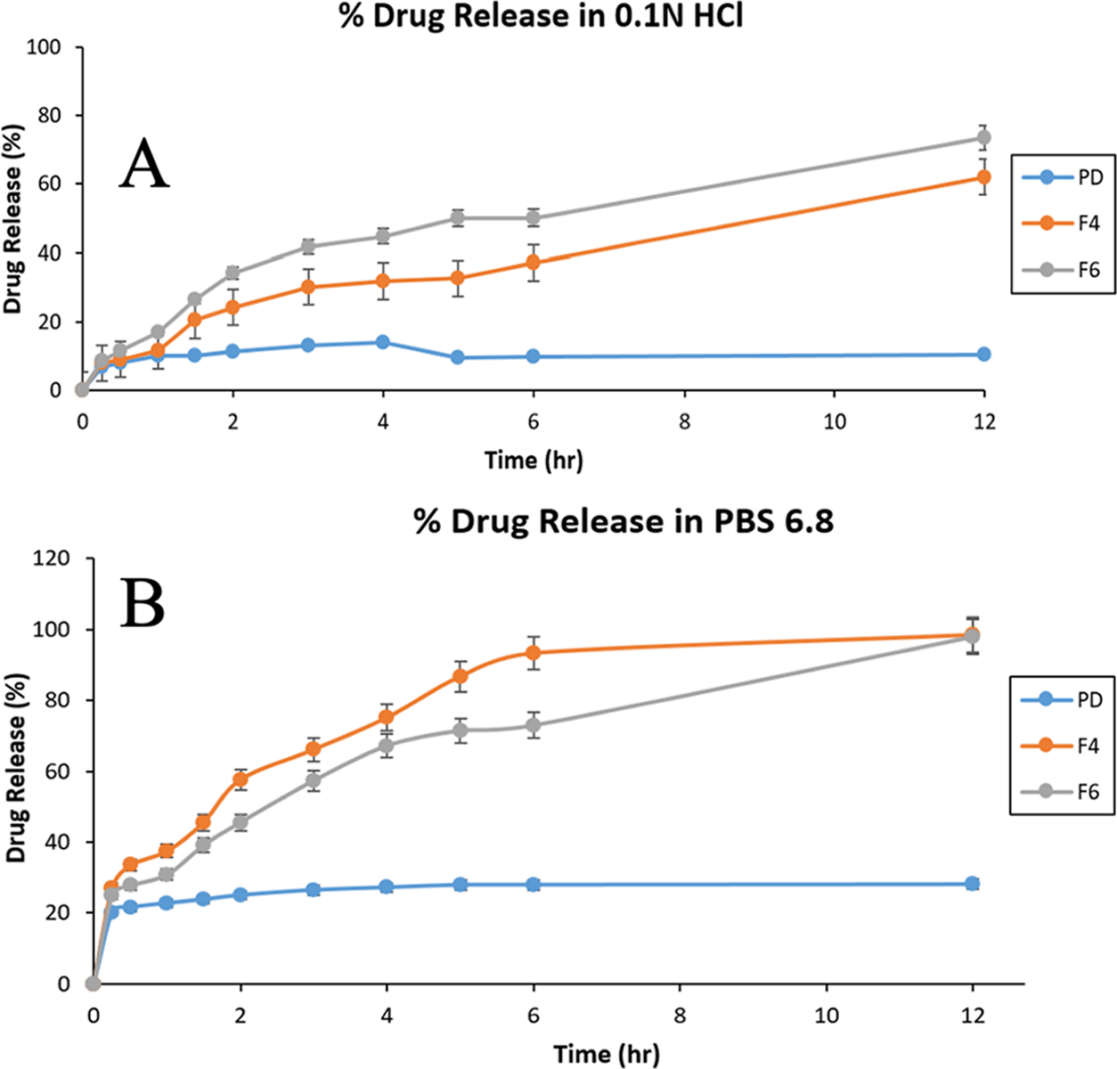
% Drug release of Pure Drug (PD), TCG-SNEDDS F4 and F6 in 0.1 N HCl (A) and PBS 6.8 (B).

The *in vitro* drug release profile of selected TCG-SNEDDS formulations F4 and F6, as well as the pure TCG suspension (control), were carried out in 0.1 N HCl and PBS 6.8 mediums at 37 °C. In this study, the drug release in 0.1 N HCl and PBS 6.8 for 12 h was assessed. Figure 12 depicts a graphical representation of the drug release profiles of TCG (pure drug suspension) and TCG-SNEDDS (F4 and F6). The *in vitro* release profiles of TCG from SNEDDS formulations F4 and F6 provided a consistently better release pattern in PBS 6.8, while the release of the drug was notably hampered in 0.1 N HCl in comparison to the pure TCG suspension, as illustrated in Figure 12. During the initial 2 h of the *in vitro* drug release study, about 24.97% of the TCG was released in PBS 6.8 from pure TCG suspension, whereas 57.59 and 45.54% of TCG was released from F4 and F6 formulations in PBS 6.8. In 0.1 N HCl, 11.26% of TCG was released from pure TCG suspension, while 24.16 and 34.18% of TCG were released from F4 and F6 formulations, respectively. TCG-SNEDDS formulation F4 displayed an improved drug release of 93.31%, and F6 exhibited 72.82% drug release, while pure TCG suspension displayed a release of 27.91% in PBS 6.8 within 6 h. On the contrary, drug release in 0.1 N HCl from pure TCG suspension, F4, and F6 were 9.66, 37.06, and 50.12% within 6 h, respectively. The formulation F4 (98.45%) showed the highest release in comparison to F6 (97.86%) and pure TCG suspension (28.05%) in PBS 6.8 after 12 h. While formulation F6 exhibited a high drug release of 73.57% as compared to F4, and pure TCG suspension released 62.03 and 10.35%, respectively. The dissolution study was extended for a duration of 24 h in order to observe and identify any potential instances of precipitation or fluctuations that may arise during this time period. Statistical comparison of formulations revealed that F4 (*p* = 0.0210) and F6 (*p* = 0.0063) were releasing the drug significantly greater in percentile as compared to the pure TCG suspension in 0.1 N HCl, while in PBS 6.8, F4 (*p* = 0.0011) and F6 (*p* = 0.0117) demonstrated significant drug release in comparison to pure TCG suspension, thereby indicating that TCG-SNEDDS formulations exhibited a significantly enhanced drug release profile in comparison to control (with the significance level of *p* < 0.05) in both dissolution media.^44^

### 3.18 *In Vitro* kinetic Drug Release Modeling

The *in vitro* release kinetics were evaluated in order to determine the most appropriate release behavior for the TCG formulations. The release data obtained *in vitro* was examined using several kinetic models, including first and zero order, as well as the Korsmeyer–Peppas model. The analysis of the kinetic modeling data indicated that TCG-SNEDDS adhered to the Korsmeyer–Peppas model, as depicted by the strong correlation coefficient values observed in both instances. After conducting a comparison of *R*^2^ values, it was determined that the Korsmeyer–Peppas model had the highest level of fit among the models considered. The data obtained demonstrated that the release of TCG-SNEDDS F4 and F6 followed a diffusion-controlled mechanism in PBS 6.8, indicating their potential as a means to achieve sustained drug release from SNEDDS formulations. Based on the observed diffusion coefficient value, denoted as n, which is less than 0.45, it can be confidently concluded that the drug released from the SNEDDS follows a Fickian diffusion process. However, the release of TCG-SNEDDS F4 and F6 in 0.1 N HCl followed an anomalous mechanism (diffusion and erosion). With the diffusion coefficient (n) above 0.45, it can be reasonably asserted that the drug released from TCG-SNEDDS in 0.1 N HCl follows non-Fickian transport. The outcomes derived from the application of several kinetic models are shown in Table 6. In this table, the kinetic constants are denoted by the symbol k.^45^

**Table 6:** Release Kinetic Studies of PD, TCG-SNEDDS F4, and F6 in PBS 6.8 and 0.1 N HCl

		PBS 6.8			0.1 N HCl		
kinetic models		PD	F4	F6	PD	F4	F6
zero order	*K*_0_	4.024	12.283	10.987	1.595	6.081	7.872
	*R*^2^	0.2151	0.0845	0.3457	0.3762	0.9622	0.9275
first order	*K*_1_	0.057	0.426	0.292	0.018	0.091	0.145
	*R*^2^	0.9241	0.9384	0.9098	0.3955	0.9800	0.9829
Korsmeyer–Peppas model	KKP	23.235	44.161	35.780	9.557	14.430	21.356
	*R*^2^	0.9938	0.9664	0.9887	0.8888	0.9901	0.9911
	*n*	0.095	0.360	0.409	0.093	0.573	0.506

### 3.19 *Ex Vivo* Drug Permeation Study

The results of the *ex vivo* drug permeation study carried out by the gut sac method are presented in Table 7. The outcomes indicated that the total amount of the drug permeated through the rabbit’s intestine was greater in F4 as compared to the pure TCG suspension. At the end of the 3 h, the apparent permeability and steady-state flux of TCG pure suspension were 0.6708 × 10^–6^ cm^2^/s and 0.012 μg/min, respectively. For TCG-SNEDDS F4, the apparent permeability and steady-state flux were 2.7 × 10^–6^ cm^2^/s and 0.066 μg/(cm^2^ min), respectively, and the amount of the drug perfused through the intestine can be arranged in the descending and sequence F4 > C. TCG-SNEDDS formulation F4 exhibited a substantially enhanced drug release pattern in comparison to pure TCG suspension.^39^ The statistical analysis unveiled a significant difference in the release of drug among the TCG-SNEDDS F4 formulation and the pure TCG suspension (*p* < 0.0001), Student’s *t* test results indicated that the drug release from TCG-SNEDDS F4 was significantly greater than the pure TCG suspension.

**Table 7:** *Ex Vivo* Drug Permeation Results of TCG Suspension and TCG-SNEDDS F4

formulations	apparent permeability coefficient *P*_app_ × 10^–6^ cm^2^/s	efflux *J* (d*Q*/d*t*) μg/min
TCG suspension	0.6708	0.012
TCG-SNEDDS F4	2.7	0.066

### 3.20 Pharmacodynamic Study

**Figure 13:**
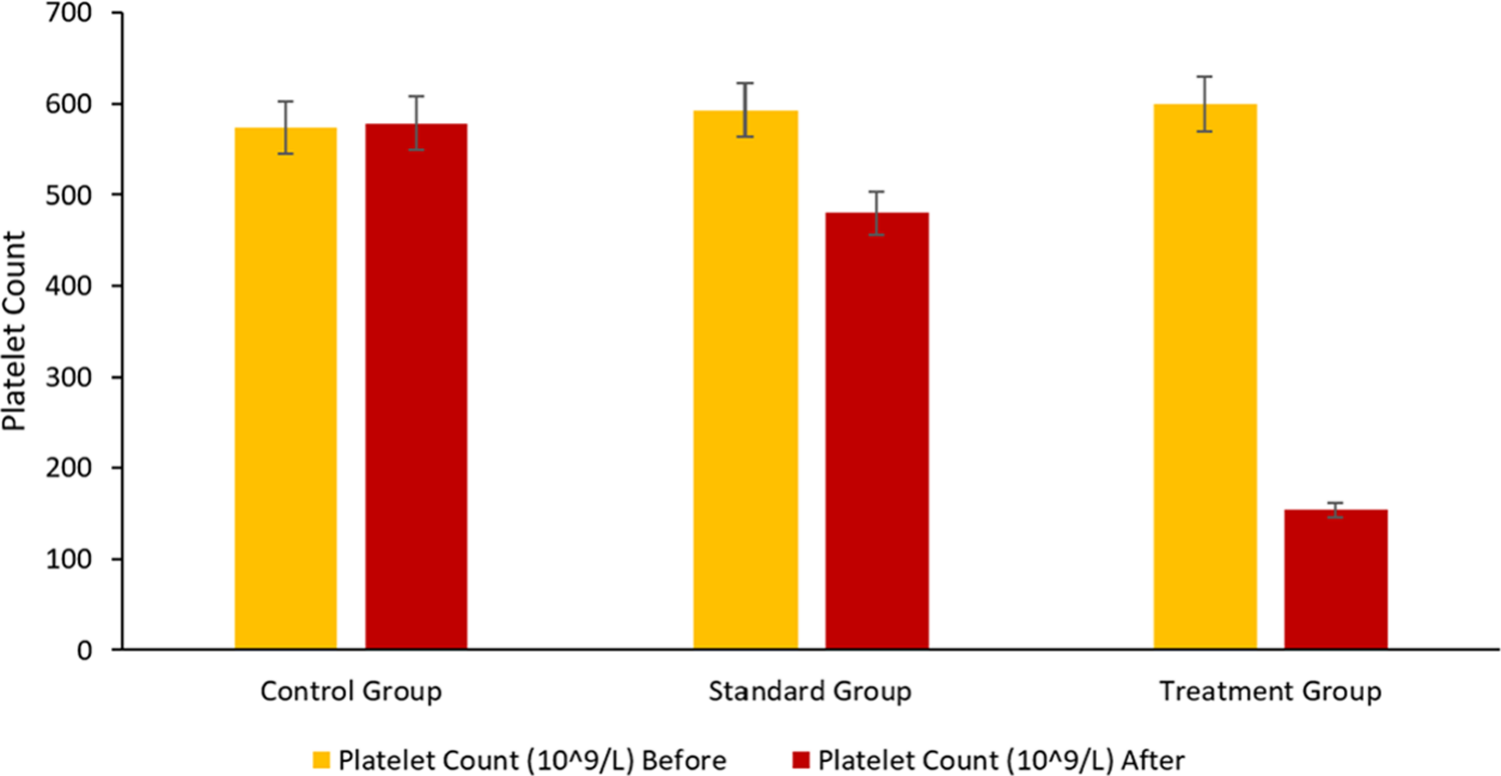
CBC chart representing mean platelet count before and after the commencement of treatment *versus* control, standard, and treatment groups.

**Figure 14:**
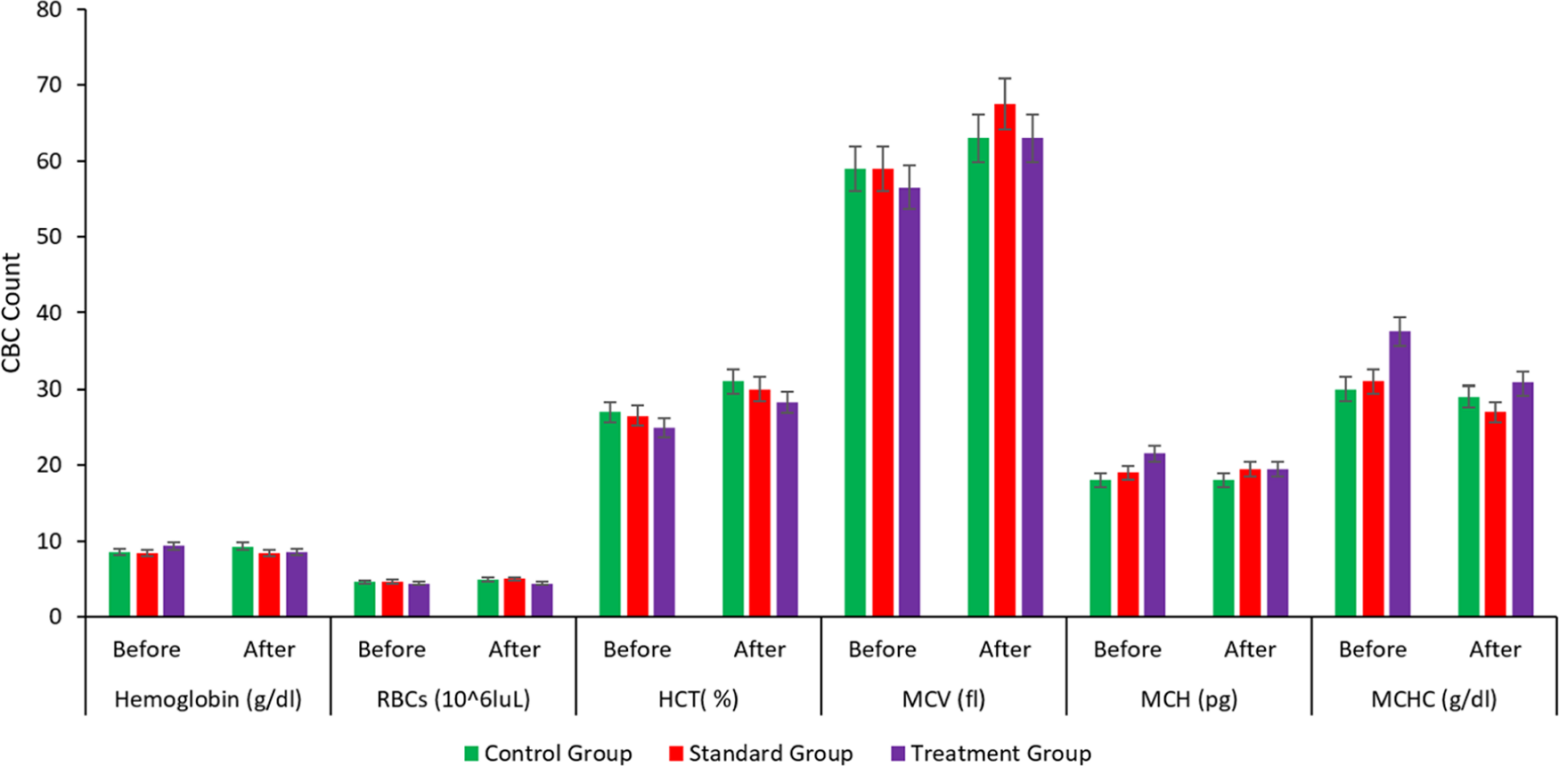
CBC parameters chart plot between hemoglobin (g/dL), RBCs (10^6^ LμL), HCT (%), MCV (fl), MCH (pg), and MCHC (g/dL) of control, standard, and treatment groups.

**Figure 15:**
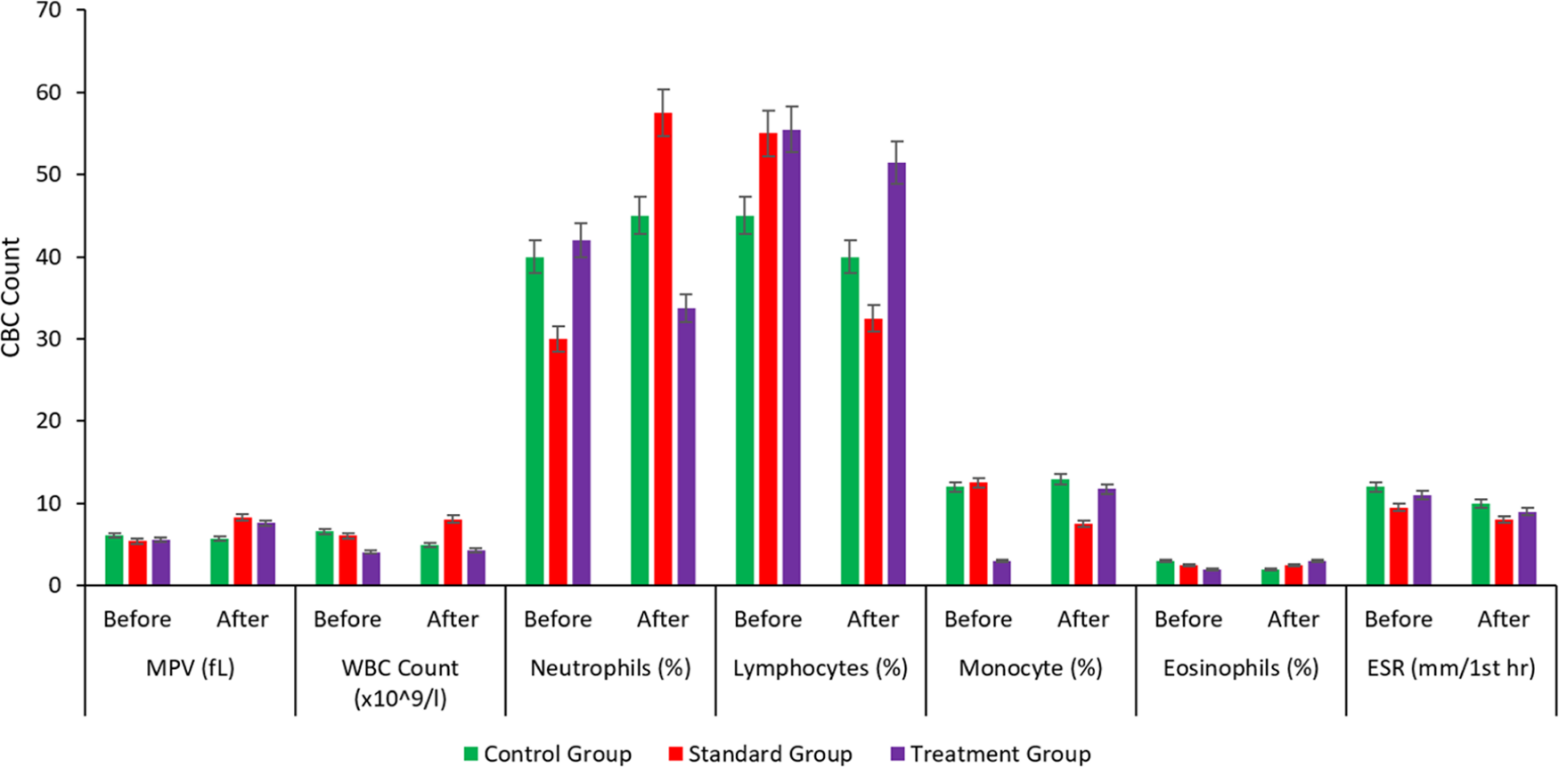
CBC parameters chart plot of MPV (fl), WBC count (×10^9^/L), neutrophils (%), monocytes (%), eosinophils (%), PDW, and ESR (mm/1st hour) between control, standard, and treatment groups.

For the purpose of evaluating the pharmacodynamics of the TCG-SNEDDS formulation F4, as well as a pure TCG suspension, an oral dosage corresponding to 8 mg/kg was given to two groups of rabbits. The Standard group received the pure TCG suspension, the Treatment group received TCG-SNEDDS F4, and the Control group was given a placebo. A complete blood count (CBC) with erythrocyte sedimentation rate (ESR) of each group was recorded before the commencement of the treatment and after the administration of respective doses for 24 h. Figure 13 reveals that the mean platelet count was significantly reduced in the treatment group as compared with the standard and control groups. The mean platelet count decrease occurred 24–48 h after the administration of TCG-SNEDDS F4 and remained within the reference laboratory range. The enhanced antiplatelet activity of TCG-SNEDDS F4 may be due to the enhanced solubility and permeability facilitated by the enhanced drug absorption and also the reduced size of TCG due to the formation of SNEDDS. The ESR of all three groups was also in the reference laboratory range, indicating no abnormal levels of inflammation in the body, as indicated in Figure 14. Other CBC parameters were also within the reference laboratory ranges, as shown in Figures 14 and 15.

## 4 Discussion

Ticagrelor, a P2Y12 platelet inhibitor, is prescribed for individuals with prior myocardial infarction (MI) or with acute coronary syndrome (ACS) to diminish the risk of future MI, stroke, and cardiovascular mortality. Unlike certain drugs, TCG is not a prodrug, remains unaffected by CYP 450 genes, and demonstrates lower interindividual variability. PLATO studies conclusively demonstrated a remarkable mitigation in mortality rate associated with TCG compared with Clopidogrel.^46^ TCG is a BCS category IV drug, indicating limited solubility and permeability. SEDDS is a lipid-based DDS that serves the dual purpose of enhancing the dissolution profile of hydrophobic drugs and shielding them from unfavorable environments within the gut. By hampering the rapid release of TCG in the stomach, SEDDS could also be employed to reduce the adverse effects of the drug in the local mucosa. The key element in crafting formulations of SEDDS with precise physicochemical attributes lies in the meticulous choice of the components. The capacity to load the drug within the SEDDS formulation is determined by the solubility of API, in this case, TCG, within the various components. This selection process relies on conducting solubility studies, which are aimed at pinpointing the most appropriate oil, surfactant, and co-surfactant combinations with the highest potential of solubilizing the drug, thus allowing optimal drug loading.^47^ Oils continue to serve as the primary constituent or excipient within the SEDDS formulation due to their capacity to enhance the solubility of hydrophobic drugs. However, the surfactants play a pivotal role in stabilizing the formulations of SEDDS, with their type as well as quantity dictating the size and stability of the emulsion globules. The nonionic surfactants are frequently favored over the strongly charged and amphiphilic surfactants, mainly due to their reduced toxicity and heightened resilience to variations in pH and ionic strength.^48^

When the outcomes for various excipients were compared, it became evident that the mixtures with a higher proportion of Tween-80 exhibited superior self-emulsifying properties. To gauge the effectiveness of TCG-SNEDDS, one can assess it by examining the rate at which emulsification occurs, a step known to influence the absorption of the drug. The findings depicted in Table 3 demonstrated that a shorter SE time signifies the formulation’s capability for swift and effortless emulsification. Moreover, these results highlight that the emulsification is influenced by the system composition and the proportion of oil to S-mix. Analyzing the SE time data led to the conclusion that the process of SE occurs spontaneously and the duration required for SE diminishes when the concentration of surfactant is increased. This implies that the TCG-SNEDDS formulations have the ability to disperse rapidly and effortlessly upon being subjected to gentle agitation.

Droplet size serves as a crucial factor in influencing both the speed and extent of drug release, which subsequently affects the absorption of the drug. Research has depicted that the smaller droplet size of the drug offers a more extensive interfacial surface area for its absorption, leading to the remarkable dissolution profile of the drug.^49^ The ζ size of TCG-SNEDDS ranged from 11.85 to 206.4 nm, demonstrating that the prepared TCG-SNEDDS formulations fall within the nanoemulsion category. Another critical parameter often used to assess particle uniformity is the polydispersity index (PDI), in the range of 0.0–0.5. The smaller value of PDI depicts a more homogeneous nanoemulsion with remarkable stability. Furthermore, ζ potential (mV) measurements of the selected TCG-SNEDDS formulations ranged from −9.92 to −6.23 mV, indicating that these formulations exhibit stability. The physiological environment of the GI tract contains a variety of ions that reduce the surface charge of nanoemulsions formed by SEDDS. Additionally, a prior investigation substantiated that the nanoparticulate fusion is favored in the stomach due to its acidic nature and elevated ionic strength.^50^ Similarly, the negatively charged layer of the gastric mucosa tends to repel the negatively charged SNEDDS formulations, leading to a shortened gastric emptying time. This abbreviated gastric emptying time results in the swift passage of formulations *via* the stomach, consequently leading to a decreased drug release in the stomach and ultimately leading to a diminished exposure to the gastric mucosa, thereby reducing the likelihood of GI distress.

The outcomes of the thermodynamic stability evaluation for SNEDDS were good, indicating robust stability even when subjected to stress conditions. The results notably revealed the absence of any signs of phase separation, crystallization, or flocculation. The determination of the cloud point holds significance in predicting the stability and precipitation tendencies of the prepared SNEDDS. It serves as an indicator of whether surfactants might precipitate at elevated temperatures. This concern arises because higher temperatures can potentially lead to the loss of water from surfactant molecules, resulting in the gelation of the formulation and the forfeiture of its emulsification properties. The cloud point determination was conducted at temperatures exceeding the physiological levels. The findings lead to the conclusion that all TCG-SNEDDS formulations could retain stability under *in vivo* circumstances. TCG-SNEDDS F4 and F6 formulations exhibited the highest cloud point.

The absorption bands seen in the FTIR spectra of TCG-SNEDDS were located to be in regions comparable to those of pure TCG. Moreover, there was no indication of interaction among the primary peaks. This suggests that the TCG drug demonstrates chemical stability when incorporated into the SNEDDS. This phenomenon can likely be attributed to the similarity in functional linkages present in the surfactant, co-surfactant, and oil phase used. SEM images also confirm the absence of oblong-rod-shaped crystalline structures of the TCG in the SNEDDS formulations verifying the successful entrapment of TCG in SNEDDS form. The results from DSC and TGA revealed that the TCG drug exists in a molecularly dissolved and amorphous state within SNEDDS formulations. Additionally, XRD analysis did not show any distinct peaks of the TCG that would typically represent the absence of the crystalline structure of TCG in the final formulation. The improvement in the observed *in vitro* drug release of TCG can be ascribed to the impromptu formation of emulsions during the dissolution procedure. Consequently, this spontaneous dissolution of the drug from TCG-SNEDDS has the potential to result in increased absorption and enhanced oral bioavailability. The strong correlation between the ζ size and *in vitro* TCG release is established. This may suggest that the immediate release of drug in the intestine is facilitated due to the greater interfacial region in emulsion.^51^ The deficient performance of TCG pure suspension can be ascribed to inferior aqueous solubility and inadequate permeability of TCG. TCG-SNEDDS formulations F4 and F6 exhibited notably enhanced release of the drug showing the highest level of significance *versus* control (*p* < 0.05). In the *ex vivo* drug permeation study, the enhanced intestinal uptake of TCG *via* the TCG-SNEDDS formulation could be rationalized in several ways. The rapid infiltration of TCG into the intestinal sac and immediate dispersion could explain the heightened level of penetration. The presence of emulsion droplets in the nano-size range within the intestinal region ultimately improves TCG absorption. Moreover, TCG-SNEDDS formulations, with elevated drug solubility and swift SE properties, likely contributed to enhanced TCG absorption within the intestine. The bioenhancing potential of Tween-80 surfactant and PEG-400 co-surfactant results in improved permeability by the disruption of lipids located in the cell membrane.^52^ The pharmacodynamic study results revealed that there was a significant decrease in the mean platelet count after the administration of TCG-SNEDDS compared to the pure TCG suspension, thereby indicating high antiplatelet activity. Hence, successful improvement in the solubility and permeability attributes of the TCG drug was observed after the incorporation into SNEDDS.

## Conclusions

The primary purpose of this research work was to develop SNEDDS of BCS class IV drug, *i.e.*, Ticagrelor with inadequate solubility and permeability. The solubility evaluation helped in the selection of appropriate excipients for the formation of SNEDDS, whereas pseudo-ternary phase diagrams added value in finalizing the suitable ratios of the formulation components, *i.e.*, Clove oil as oily phase, Tween-80 employed as a surfactant, and PEG-400 as a co-surfactant in the ratios of 10–20, 45–70, and 20–45%, respectively. The selected formulation exhibited nanosized droplets having a negative charge on the surface, significantly improved release and permeation of the drug that has advocated the usefulness of the SNEDDS for BCS-IV drugs.
